# Supplementary material for: Solar radiation model and optimization of asymmetric large-span externally insulated plastic greenhouses
Source: PLoS One. 2025 Jan 7;20(1):e0309734. doi: 10.1371/journal.pone.0309734 (PMC11706437; doi:10.1371/journal.pone.0309734)
Supplement: Attachment — Submitted filename: Response to Reviewers.docx [file pone.0309734.s003.docx]

Dear reviewer,

Thank you very much for your hard work and patience. We have carefully revised the manuscript based on your comments, and the following is a response to the comments.

Reviewer #1: The authors have satisfied most of my queries, still I do not see nephographs or false colour maps of the distribution of radiation in plan inside the greenhouse

Answer: Thank you for the advice. Critten's findings suggest that the length has little influence on the greenhouse's light transmission in the east-west direction [1]. Hence, this study treats the greenhouse length as infinite and focuses only on how its span affects the light environment. According to the solar radiation model, the spatial distribution of noon solar radiation for different structures of greenhouses on the winter solstice can be seen in Figure 8 (line 539).

**Fig 8. Spatial distribution of different structured greenhouses at noon on the winter solstice.**

**(a): ridge height 5.3, 12m+8m;** **(b): ridge height 5.3, 11m+9m; (c): ridge height 5.8, 12m+8m; (d): Ridge height 5.8, 11m+9m.**

 Reviewer #3: Authors have addressed my review comments successfully. However, the following comment was not addressed-The novelty of the greenhouse design compared with existing designs may be highlighted.

Answer: Following your helpful suggestions, the innovation of optimizing greenhouses compared with existing greenhouses is summarized as follows: This study proposes a new solar radiation model incorporating the projection path equations of the insulation quilts, combined with a multi-objective optimization method to optimize the structural parameters of large-span plastic greenhouses suitable for Jinan, China. The results indicate that the optimal greenhouse configuration features a span of 20 meters, a height of 6.97 meters, and a north-facing roof projection length of 7.44 meters. Compared to the initial greenhouse, cumulative radiation increased by 3.37%, and light distribution uniformity significantly improved. Please kindly refer to lines 605-615 and 641-647 for the details.

**References**

1.	Critten DL. The effect of house length on the light transmissivity of single and multispan greenhouses. J Agric Eng Res. 1985;32(2):163-72.

We appreciate your consideration and constructive comments on our manuscript and we look forward to hearing from your positive answer.

With our sincere respects.

Dr. Pro. Min Wei

Dr. Chuanqing Wang
